# CRISPR/Cas9-Based Cellular Engineering for Targeted Gene Overexpression

**DOI:** 10.3390/ijms19040946

**Published:** 2018-03-22

**Authors:** Mark J. Osborn, Christopher J. Lees, Amber N. McElroy, Sarah C. Merkel, Cindy R. Eide, Wendy Mathews, Colby J. Feser, Madison Tschann, Ron T. McElmury, Beau R. Webber, Chong Jai Kim, Bruce R. Blazar, Jakub Tolar

**Affiliations:** 1 Department of Pediatrics, Division of Blood and Marrow Transplantation, Medical School, University of Minnesota, Minneapolis, MN 55455, USA; leesx002@umn.edu (C.J.L.); leira001@umn.edu (A.N.M.); niel0216@umn.edu (S.C.M.); eidex007@umn.edu (C.R.E.); mathe034@umn.edu (W.M.); feser004@umn.edu (C.J.F.); mcelm001@umn.edu (R.T.M.); blaza001@umn.edu (B.R.B.); tolar003@umn.edu (J.T.); 2 Stem Cell Institute, University of Minnesota, Minneapolis, MN 55455, USA; tscha034@umn.edu (M.T.); webb0178@umn.edu (B.R.W.); 3 Center for Genome Engineering, University of Minnesota, Minneapolis, MN 55455, USA; 4 Institute for Engineering in Medicine, University of Minnesota, Minneapolis, MN 55455, USA; 5 Asan-Minnesota Institute for Innovating Transplantation, University of Minnesota, Minneapolis, MN 55455, USA; ckim@amc.seoul.kr; 6 Department of Pediatrics, Division of Hematology, Oncology, and Transplantation, Medical School, University of Minnesota, Minneapolis, MN 55455, USA; 7 Asan Institute for Life Sciences, Asan Medical Center, Seoul 138-736, Korea

**Keywords:** CRISPR/Cas9, recessive dystrophic epidermolysis bullosa, transcriptional activation, homology directed repair, adeno-associated virus, ubiquitous chromatin opening element, T-cells, cord blood

## Abstract

Gene and cellular therapies hold tremendous promise as agents for treating genetic disorders. However, the effective delivery of genes, particularly large ones, and expression at therapeutic levels can be challenging in cells of clinical relevance. To address this engineering hurdle, we sought to employ the clustered regularly interspaced short palindromic repeats (CRISPR)/Cas9 system to insert powerful regulatory elements upstream of an endogenous gene. We achieved robust activation of the *COL7A1* gene in primary human umbilical cord blood CD34^+^ hematopoietic stem cells and peripheral blood T-cells. CD34^+^ cells retained their colony forming potential and, in a second engineering step, we disrupted the T-cell receptor complex in T-cells. These cellular populations are of high translational impact due to their engraftment potential, broad circulatory properties, and favorable immune profile that supports delivery to multiple recipients. This study demonstrates the feasibility of targeted knock in of a ubiquitous chromatin opening element, promoter, and marker gene that doubles as a suicide gene for precision gene activation. This system merges the specificity of gene editing with the high level, sustained gene expression achieved with gene therapy vectors. We predict that this design concept will be highly transferrable to most genes in multiple model systems representing a facile cellular engineering platform for promoting gene expression.

## 1. Introduction

Recessive dystrophic epidermolysis bullosa (RDEB) is a prototypical recessive condition and is characterized by mutations to the *COL7A1* gene on chromosome 3. *COL7A1* is likewise a prototypical large gene and spans ~31 kb and contains 118 exons with an open reading frame of ~9 kb [1,2]. RDEB causative mutations occur over the span of the gene and the resultant phenotype is characterized by diminished/absent type VII collagen (C7) protein causing mucocutaneous disease manifestations. Severe, chronic skin blistering occurs along with esophageal strictures, mitten deformities, dental anomalies, corneal scarring, and increased incidence for aggressive squamous cell carcinomas [3]. Therapeutic benefit can be achieved by the delivery of functional C7 protein. Sources of C7 include transplant of allogeneic or gene corrected autologous cells and/or recombinant C7 protein injection.

Woodley and colleagues delivered recombinant C7 protein by intravenous injection showing that C7 produced locally or from a distance can mediate a functional benefit [4]. However, repetitive injections of recombinant peptide over the course of a patient’s lifetime are fiscally burdensome, making cellular sources an attractive option. Allogeneic cellular injections have resulted in improved skin integrity; however, the low expression levels of *COL7A1* from the endogenous promoter results in poor delivery beyond the site of injection [5]. Further, allogeneic cells may not persist long term due to host immune-mediated clearance [6]. Autologous cellular engineering is highly promising due to the lowered risk of immune rejection, and *COL7A1* gene expression has been restored in patient derived cells using gene therapy and gene editing [7,8].

To encode, deliver, and express *COL7A1*, gamma retroviral and lentiviral expression vectors have been developed and deployed that result in supraphysiological *COL7A1* gene expression. However, the large size of the *COL7A1* cDNA can result in lowered titers that can make effective delivery a challenge [5,9,10,11,12]. Efforts have been undertaken to use less size-restricted platforms such as the phiC31 integrase, or Sleeping Beauty, transposon; however, the effective delivery of these vectors can similarly be challenging [5,13,14]. Additionally, the semi-random genomic integration profiles of these systems in the premalignant RDEB phenotype represents a significant safety concern due to insertional mutagenesis [15,16,17].

To capitalize on the precise targeting capabilities afforded by gene editing, we have targeted the *COL7A1* gene with transcription activator like effector nucleases (TALEN) and the clustered regularly interspaced short palindromic repeats (CRISPR)/Cas9 system derived from *Streptococcus pyogenes* [8,18]. Along with zinc finger nucleases and meganucleases, TALENs and CRISPR/Cas9 represent programmable reagents capable of generating single or double stranded DNA breaks at user-defined loci [19,20]. This stimulates homology directed repair (HDR) from an exogenous template allowing for precision genome modification. In situ gene correction maximizes safety but gene control is regulated by the comparatively weak *COL7A1* promoter. As such, the systemic therapeutic impact may be incomplete due to the limited distribution of C7 protein.

We hypothesized that we could synergize the attributes of gene therapy and gene editing: supraphysiological gene expression and a high degree of specificity. Previous efforts to accomplish this have centered on “safe harbor” site incorporation of a candidate gene driven by exogenous regulatory elements [21]. Delivering a cargo as large as the ~9 kb *COL7A1* cDNA can be challenging making this approach sub-optimal. To address this, we devised a strategy whereby we could incorporate a powerful transcriptional activator into the native *COL7A1* locus. This resulted in profound upregulation of the endogenous *COL7A1* gene. Because our approach relies on a functional gene embedded in the genome, we pursued our strategy in cells with a favorable immunological profile in that they either innately, or can be engineered to, have a low frequency and incidence of immune-based side effects. Umbilical cord blood (UCB) derived hematopoietic stem cells (HSC) are effective for allogeneic therapy and display reduced rates of graft versus host disease (GVHD) [22,23]. Here we show robust *COL7A1* gene activation in UCB HSCs with maintenance of their multi-lineage differentiation potential in colony forming assays. In parallel, we pursued T-cell engineering and observed *COL7A1* expression levels that surpassed those of wild type keratinocytes. By subsequently ablating the T-cell receptor complex we generated a stable population of T-cells with a low risk of triggering GVHD. Collectively, our engineering approach allows for targeted gene upregulation with HDR dependent co-expression of a clinically relevant cell surface marker that serves as a selection agent and suicide gene. This strategy represents a streamlined translational engineering platform readily and rapidly transferrable to multiple model systems for user-defined transcriptional modification.

## 2. Results

### 2.1. COL7A1 Promoter Targeting for Transcriptional Activation

**Figure 1:**
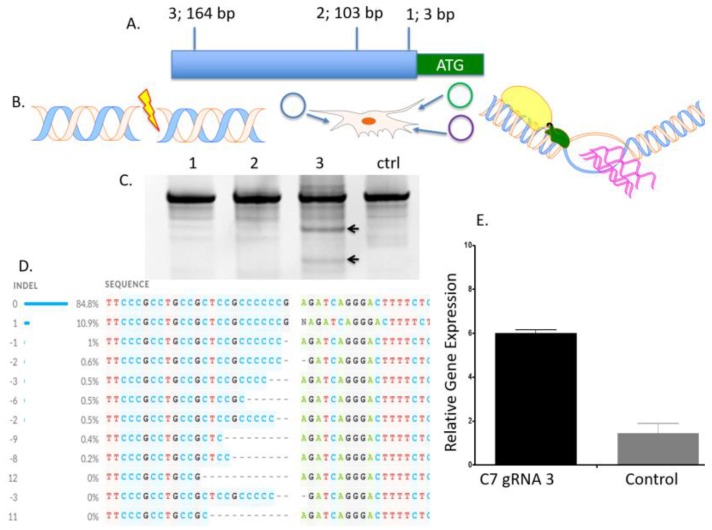
*COL7A1* promoter targeting and transcriptional activation. (**A**) Three guide RNAs in the *COL7A1* promoter were generated and their distance from the initiating methionine is shown. (**B**) Experimental schema. Guide RNAs were tested in HEK 293T cells for insertion deletion formation using the nuclease version of Cas9. Cas9 was delivered in cis with a guide RNA (gRNA) as plasmid DNA (blue circle) and the DNA helix at left diagrams a nuclease induced double stranded DNA break. At right is the diagram for gene activation assessment. A catalytically inactive Cas9 fused to a VP64 transcriptional domain was delivered in trans with a plasmid gRNA (green and purple circles, respectively). The Cas9-VP64 transcriptional activation domain (green oval) mediates gene upregulation and pink lines indicate mRNA transcripts. (**C**) Surveyor nuclease assay. Genomic DNA was analyzed using the Surveyor method 72 h post gene transfer. Arrows indicated cleavage products. (**D**) ICE (Inference of CRISPR edits) analysis. Sanger sequence was analyzed for insertions and deletions (indel) using the ICE algorithm. Dashed lines indicate indels and at left are the numbers of bases added or deleted. Note that the gRNA sequence is in the reverse complement (**E**) CRISPR/Cas9 *COL7A1* transcriptional activation. *COL7A1* transcription levels were measured by quantitative reverse transcription-polymerase chain reaction (qRT-PCR) and fold increase above untreated cells is shown (*n* = 3 for all experiments).

**Figure A1:**
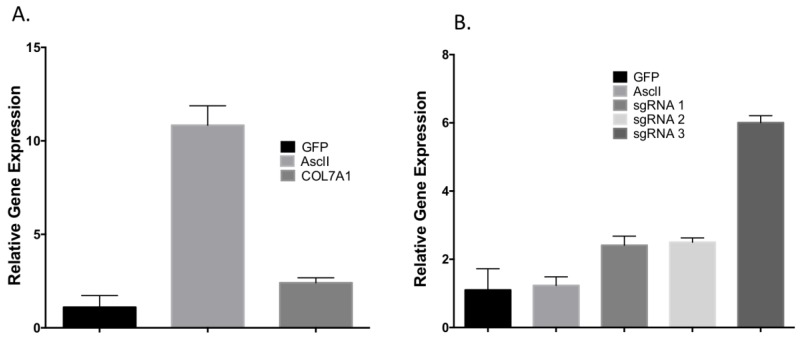
HEK 293T cells were lipofected with a catalytically inactive Cas9-VP64 plasmid and GFP (negative control) and candidate gRNAs. (**A**) Positive control. HEK 293T cells were transfected with GFP, a positive control gRNA (AsclI), and *COL7A1* gRNA candidate three. At 72 h the cells were analyzed for AsclI gene expression. (**B**) *COL7A1* candidate testing. The three *COL7A1* gene candidate gRNAs, an AsclI gRNA, and a GFP transfection control were delivered to HEK 293T cells. *COL7A1* gene expression was assessed at 72 h post gene transfer.

Three guide RNA (gRNA) candidates in the *COL7A1* gene immediately upstream of the start codon were generated for testing in human embryonic kidney (HEK) 293T cells (Figure 1A,B). The gRNAs were first assessed for activity by the Surveyor method and cleavage products consistent with nuclease activity were observed (Figure 1C). The insertion and deletion (indel) profile for the most active candidate (gRNA 3) was mapped using Inference of CRISPR Edits (ICE) and showed indels consistent with CRISPR/Cas9 cutting and error prone DNA repair [24]. This gRNA was then tested for its ability to upregulate endogenous *COL7A1* gene activity using a nuclease inactive Cas9 fused to a VP64 transcriptional activation domain [25] that resulted in a sixfold upregulation over untreated cells (Figure 1E). Consistent with the ICE data, testing of gRNAs 1 and 2 in the gene activation assay did not show significant *COL7A1* gene upregulation (Figure A1). These data show that gRNA candidate three’s proximity to the start codon is optimal for modulating *COL7A1* transcription and served as the targeting site for incorporation of a transcriptional activator payload.

### 2.2. Gene Editing Optimization of Human Umbilical Cord Blood Derived Hematopoetic Stem Cells

**Figure 2:**
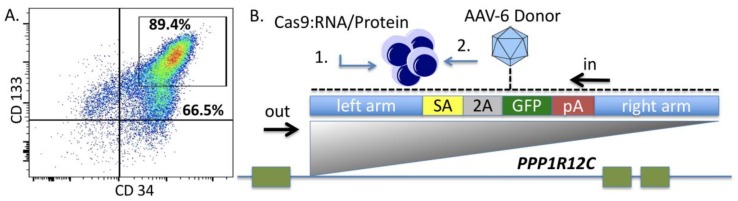

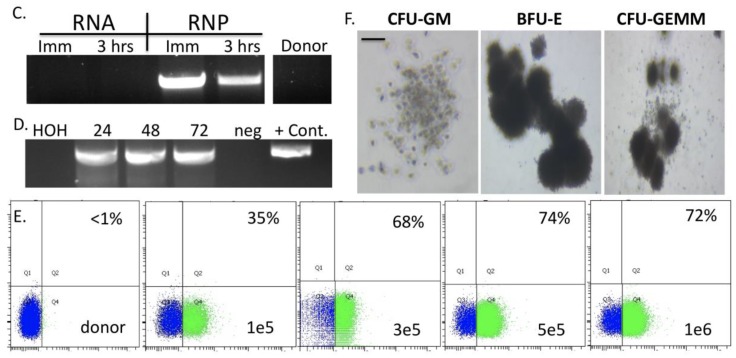
Genome editing optimization in human hematopoietic stem and progenitor cells. (**A**) Hematopoietic stem cell phenotyping. CD34 cells were isolated from cord blood and analyzed for CD34 and CD133, markers of hematopoietic lineage stem cells; (**B**) Gene editing experimental schema. Cas9 was delivered, via electroporation, to human cord blood derived CD34^+^ cells as mRNA with a guide RNA with phosphorothioate modifications or as a recombinant peptide with a gRNA without modification. Adeno-associated virus (AAV) serotype 6 was used to deliver a homology directed repair template for the *PPP1R12C* gene on chromosome 19. The homologous recombination repair reporter is shown in relation to the *PPP1R12C* target locus. The donor contains donor arms to the *PPP1R12C* locus that flank a splice acceptor (SA), 2A sequence, green fluorescent protein gene (GFP), and the bovine growth hormone polyadenylation signal (pA). Insertion into the first intron results in splicing into the reporter with subsequent GFP expression. Green boxes represent exons. (**C**) Molecular readout for homologous recombination. AAV-6 donor was added to cells that were electroporated with Cas9 mRNA or protein immediately after gene transfer (imm) or 3 h post electroporation. HDR analysis was done at 72 h using an “inside-out” PCR with one primer inside the donor and the second located at the endogenous locus (arrows in (**B**)). (**D**) Homology directed repair temporal kinetics. Cas9 RNP was electroporated into CD34 cord blood cells and AAV-6 donor (multiplicity of infection (MOI) 5e5 GC/mL) was added immediately after gene transfer. Cells were screened for HDR by allele specific inside/out PCR at 1, 2, or 3 days after donor delivery. (**E**) HDR donor template dose optimization. Various donor concentrations were added as AAV-6 particles at the indicated multiplicity of infection (lower right quadrant of FACS plot). At 72 h post modification flow cytometry was performed to assess GFP expression from the donor. Percentage of GFP positive cells are shown in each box. (**F**) Representative images of MethoCult colony forming unit assay. CFU-GM = granulocyte/macrophage, BFU-E = burst forming unit-erythroid CFU-GEMM = granulocyte, erythrocyte, monocyte, megakaryocyte. All data are representative of at least three experiments. HOH = water; neg = negative control, untreated cells; +Cont = K562 cells that were generated for use as a positive control. Black bar is 100 microns and photomicrographs were taken at 400× magnification.

**Figure A2:**
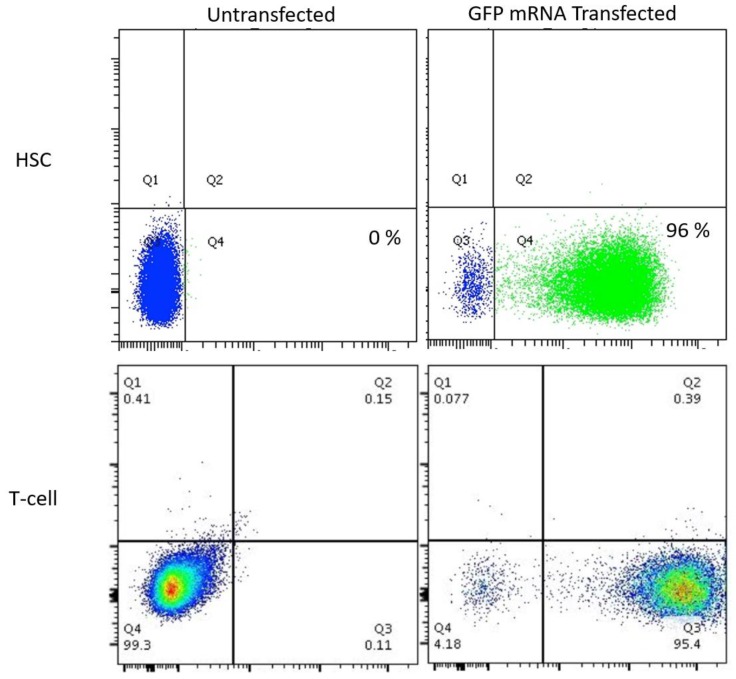
CD34 cells and T-cells were electroporated with one microgram of GFP mRNA under the optimal conditions and assessed for GFP expression at 48 h post gene transfer. Untransfected cells and a representative FACS plot of four electroporation experiments is shown.

Because bone marrow transplantation has emerged as a major treatment option for RDEB we sought to determine whether hematopoietic stem cells (HSC) were permissive to *COL7A1* targeting and gene upregulation. Human cord blood units were obtained and after CD34 cell positive selection a fluorescence activated cell sorting (FACS)-based phenotyping was performed that showed a high prevalence of CD34^+^CD133^+^ cells that are markers of HSCs (Figure 2A). To optimize gene editing in this population we first optimized the electroporation conditions and were able to consistently achieve ~95% gene transfer efficiencies (Figure A2). To optimize HDR we employed a homology directed repair (HDR) reporter that has been well characterized for the *PPP1R12C* gene on chromosome 19 [26]. The donor is designed to express green fluorescent protein only upon proper gene targeting via HDR and consists of a splice acceptor, 2A sequence, and a GFP gene (Figure 2B). We compared Cas9 mRNA or recombinant protein and donor templates encapsulated in adeno-associated virus (AAV) serotype 6 particles for their ability to mediate HDR. Cas9 mRNA and a gRNA with phosphorothioate modifications [27] or recombinant Cas9 peptide and a gRNA as a ribonucleoprotein (RNP) complex were electroporated into CD34 cells followed by AAV-6 donor addition immediately or three hours after electroporation. We observed, using an inside/out PCR screening method, that Cas9 RNP resulted in demonstrable HDR when the donor was added within a short period of time after gene transfer (Figure 2C). Because sustained culture of primary cells can negatively impact their functionality, we next assessed the kinetics of HDR by performing a time course analysis. Temporal inside/out PCR analysis showed that HDR occurs within twenty-four hours (Figure 2D). We next optimized the donor dose to determine the multiplicity of infection (MOI) best suited for CD34 cell modification. We achieved greater than 70% *AAVS1*/*PPP1R12C* locus HDR rates and peak HDR occurred at an MOI of 5 × 10^5^ genome copies/mL (GC/mL) (Figure 2E). To demonstrate that the UCB CD34 cells subjected to Cas9 RNP electroporation and AAV-6 donor transduction retained their functional properties, colony forming unit assays (CFU) were performed. Colonies with multi-lineage differentiation capabilities were observed (Figure 2F). These data showed that the optimal conditions for HDR that we defined using Cas9 RNP and AAV-6 resulted in a cellular product that maintained their ability to form blood derivatives in CFU assays.

### 2.3. COL7A1 Gene Upregulation in Primary CD34 Cells from Cord Blood

**Figure 3:**
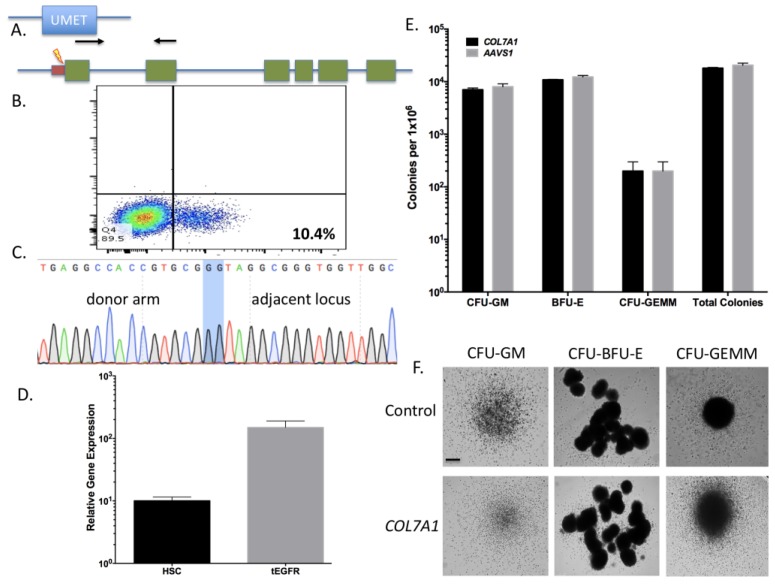
Hematopoietic cell engineering with transcriptional activation cassette knock in. (**A**) *COL7A1* targeting donor. Arms of homology were constructed for the transcriptional start site (TSS) proximal region. The left arm contained 500 bp of sequence upstream of gRNA candidate three. The right donor arm began immediately after the TSS and extended 500 bp downstream. In between the donor arms was the UMET element: a ubiquitous chromatin opening element (UCOE) and a hybrid promoter (MND). Downstream of the promoter is the truncated non-signaling epidermal growth factor receptor (tEGFR) and a 2A peptide sequence that is in frame with the endogenous *COL7A1* exon 1. Arrows are the inside/out PCR primers used for HDR detection. The donor is shown in relation to the endogenous locus. The small maroon box is the *COL7A1* 5’ untranslated region and the green boxes are the first six exons of *COL7A1*. (**B**) Donor derived tEGFR expression. Following electroporation and AAV transduction the HSCs were assessed for locus modification by tEGFR expression using flow cytometry. (**C**) *COL7A1* locus HDR. Cas9 RNP and AAV-6 donor treated cells were screened using inside/outside PCR using primes noted with black arrows in (**A**). Shaded region shows donor:endogenous locus junction. (**D**–**F**) Engineered HSC characterization. (**C**) HSCs were modified with Cas9 RNP and AAV-6 donor and assessed for tEGFR expression that is shown on the *x*-axis. (**D**) Modified HSC were sorted for tEGFR expression and analyzed for COL7A1 by qRT-PCR. Purified cells were analyzed for *COL7A1* gene expression and were compared to untreated cells (*n* = 3 experiments). tEGFR positive cells were analyzed for CFU potential (*n* = 4 donors). (**E**) CFU colony counts. (**F**) Representative photomicrographs of the multi-lineage colonies. Black bar equals 100 microns.

The transcriptional activator used in Figure 1 to validate gRNA 3 is comprised of the full length Cas9 and the VP64 activator region [25] and with this system only modest rates of *COL7A1* upregulation were obtained (Figure 1E). To drive higher rates of gene expression we devised a strategy reliant on homology directed repair to precisely insert powerful regulatory elements into the transcriptional start region of *COL7A1*. The donor targeting template had *COL7A1* gene homologous sequences flanking a ubiquitous chromatin opening element (UCOE), the MND promoter, truncated, non-signaling epidermal growth factor receptor gene (tEGFR), and a 2A sequence (Figure 3A). The UCOE prevents gene silencing and methylation and the MND is a powerful promoter that is active in lymphohematopoietic cells [28,29]. The tEGFR contains a start codon and the 2A sequence is in frame with the endogenous *COL7A1* gene. Importantly, the right donor arm lacks the native *COL7A1* start codon and gene expression is brought under the control of the initiating methionine in tEGFR upon proper targeting (Donor sequences are shown in Appendix E). Using the conditions identified in Figure 2, we electroporated UCB CD34^+^ HSCs and added AAV-6 serotype particles bearing the *COL7A1* donor. Bulk population cell screening showed ~10% tEGFR expression (Figure 3B) and gene conversion at the molecular level using an inside/out PCR methodology (Figure 3C). A qRT-PCR of tEGFR purified cells showed a 15-fold increase in *COL7A1* gene expression over unmodified HSCs (Figure 3D). In order to show that the HSC population retained its multi-lineage commitment properties, CFU assays were performed and showed a normal distribution and morphology of colonies (Figure 3E,F). These data show that HSCs can be modified for *COL7A1* gene modulation and that upregualtion does not impair lineage commitment.

### 2.4. Human T-Cell Engineering

**Figure 4:**
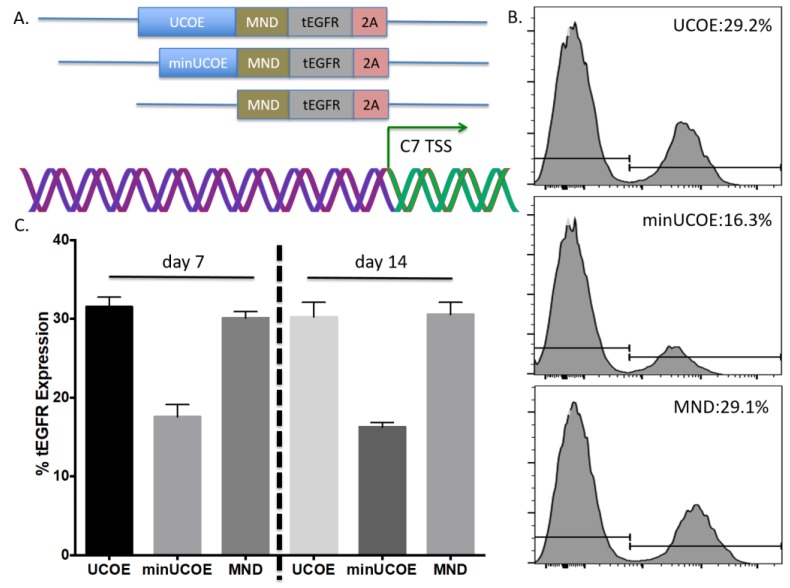
*COL7A1* promoter targeting with transcriptional promoting elements for T-cell engineering. (**A**) Transcriptional activator candidates. Diagrammed are candidates with a ubiquitous chromatin-opening element (UCOE), a truncated UCOE, or a promoter only element (MND). TSS refers to the transcriptional start site of the *COL7A1* initiating methionine and green arrow indicates direction of transcription. Purple helices in the DNA diagram indicate 5′ untranslated region and green indicate exon 1. In all configurations the left and right donor homology arm sequences were the same and each donor is constructed such that the tEGFR is in frame with *COL7A1*. (**B**,**C**) Gene modification rates. Cas9 RNP and equal MOIs of the three donors were added to primary T-cells and tEGFR expression was assessed 72 h later. Representative FACS plots are shown (**B**) and aggregate tEGFR from four replicates is shown in (**C**) at 7 or 14 days after genome modification.

To further define the optimal gene targeting constituents we tested a minimal UCOE element [30] and a donor that lacks a UCOE and had only the MND promoter (Figure 4A). Following electroporation optimization of peripheral blood T-cells (Figure A2) we tested the delivery of each donor candidate at identical MOIs. The larger UCOE containing element and MND promoter only donors resulted in the highest rates of HDR as determined by tEGFR expression (Figure 4B). Expression was maintained at a steady state level over the 14-day period in which the cells were propagated (Figure 4C). Because UCOE elements are predicted to result in more pronounced and sustained gene expression levels we employed it to determine the maximal achievable *COL7A1* locus modification rates. By increasing the donor MOI to 1 × 10^6^ GC/mL we observed ~60% gene targeting rates (Figure A4). These data show robust *COL7A1* gene targeting in human T-cells.

### 2.5. COL7A1 Modified Human T-Cell Characterization

**Figure 5:**
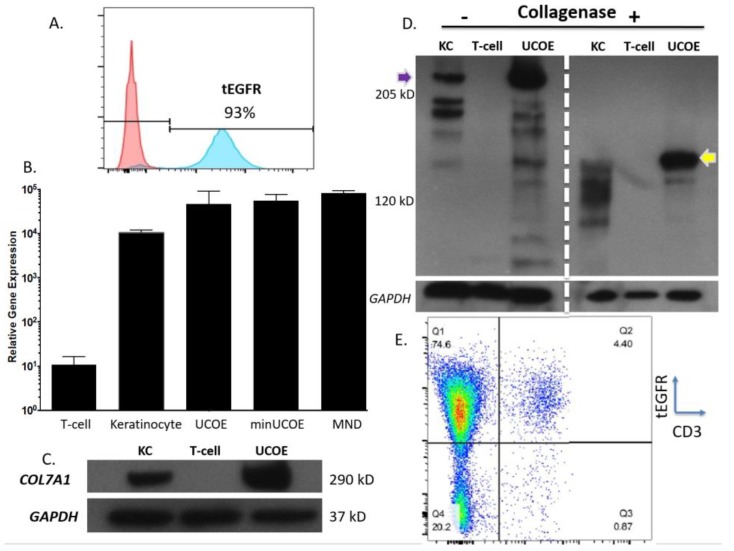
*COL7A1* promoter targeted T-cell characterization. (**A**) tEGFR-based cell purification. Cells were purified by magnetic sorting to a purity of >90% (blue portion of histogram). Pink part of FACS plot shows negative, unstained cells. (**B**) Transcriptional activation rates. Cells with >90% tEGFR purity were analyzed by qRT-PCR for *COL7A1* expression. Controls were un-manipulated T-cells and wild type keratinocytes. *p* = 0.08 for UCOE vs. minUCOE, *p* = 0.03 for minUCOE vs. MND, and *p* = 0.69 for UCOE vs. MND. *n* = 3–4 for all analyses. (**C**,**D**) Protein analysis. Lysates were taken from UCOE modified cells for Western blot with an anti-C7 antibody that detects full length peptide (**C**). Lysates were subjected to collagenase digestion that fragments the peptide into the constituent non-collagenous domains and the yellow arrow shows the 145 kD NC1 domain. Purple arrow shows the full length ~270 kD peptide. Loading controls are *GAPDH* for each. Wild type keratinocytes (KC) were the positive control and untreated T-cells were the negative control. Molecular weight marker indications are shown at left. (**E**) TRAC gene disruption. A second engineering step with electroporation of a *TRAC* gRNA Cas9 RNP resulted in ~95% TCR loss (*x*-axis is CD3 antibody staining) with >80% tEGFR (*y*-axis). FACS plot is representative of three experiments. Densitometry was performed on *n* = 3 gels for figure C and averaged 2.7 fold ± 0.5).

**Figure A3:**
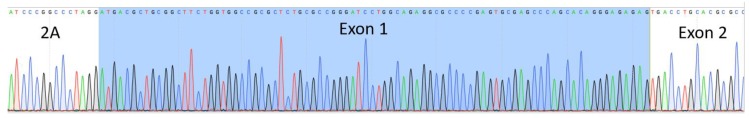
cDNA was amplified using an EGFR primer and a *COL7A1* exon 2 primer that is outside the donor arm. 2A sequence (partial) is shown at left, exon 1 is shaded in blue, and exon 2 is indicated.

**Figure A4:**
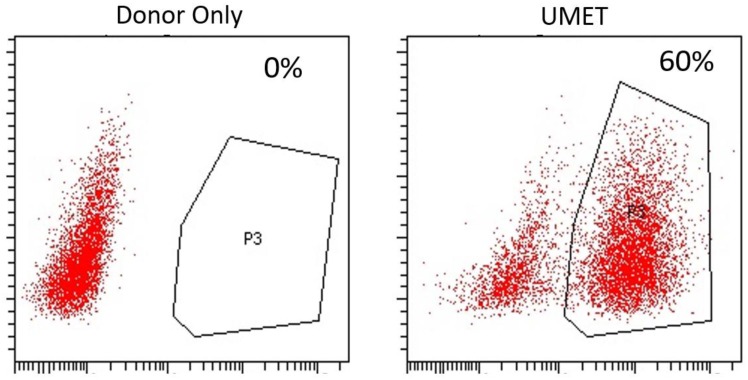
At left are T-cells that received an MOI of 1 × 10^6^ GC/mL of the UMET donor alone. At right Cas9 RNP followed by UMET donor AAV-6 transduction resulted in ~60% HDR rates. FACS plot is representative of five experiments and shows cells stained with an EGFR antibody. Gate labeled P3 shows positive cells.

To obtain a purified population of T-cells we used magnetic bead enrichment for tEGFR expression and routinely recovered >90% tEGFR positive cells (Figure 5A). qRT-PCR in tEGFR positive cells were largely equivalent between the three donor configurations in the homogeneous tEGFR positively selected cells (Figure 5B). Comparisons to unmodified T-cells and wild type keratinocytes showed that the engineered T-cells expressed ~3.5-fold higher levels of *COL7A1* mRNA than did normal keratinocytes (Figure 5B). We confirmed the presence of tEGFR: *COL7A1* fusion transcripts in isolated RNA using a primer specific for tEGFR and a second in exon 2 that is outside the donor construct (Figure A3). To show that the qRT-PCR data translated to an increase in C7 peptide production, Western blot analysis was performed on cell lysates of keratinocytes, unmodified T-cells, and UCOE-MND-tEGFR modified T-cells. Densitometric analyses showed an increase in C7 peptide levels with an average of 2.7 fold that are commensurate with the keratinocyte: engineered T-cell mRNA ratios in the qRT-PCR data (Figure 5B,C). To further confirm the structural integrity of C7 protein, the cell lysates were subjected to collagenase digestion. *COL7A1* has two non-collagenase (NC) domains, NC-1 and NC-2 [11,31] and collagenase treatment of engineered T-cell lysates showed the detection of the proper 145 kD NC-1 domain (Figure 5D). Toward further optimizing HDR in T-cells, we treated cells with a higher MOI of AAV-6 and observed maximal HDR rates of >60% (Figure A4). These data show that robust rates of HDR occur in T-cells and that they can be engineered to produce greater levels of *COL7A1* than wild type keratinocytes—one of the major *COL7A1* expressing cell populations. Finally, because allogeneic T-cells can initiate graft versus host disease (GVHD) we included a second engineering step to render the T-cells immunologically inert. This was accomplished by introducing a gRNA for the alpha chain of the T-cell receptor (TCR) that resulted in a highly pure population of EGFR positive TCR negative cells (Figure 5E) that are not predicted to trigger GVHD.

## 3. Discussion

Here we report a strategy for targeted gene upregulation using the CRISPR/Cas9 system for homology directed repair mediated insertion of a transcriptional promoting element we termed UMET. This powerful platform employs a ubiquitous chromatin opening element, the MND promoter, and translationally relevant EGFR reporter to drive endogenous gene overexpression. As proof of principle, we targeted the *COL7A1* gene that serves as a surrogate for genetic conditions involving large open reading frames that are difficult to deliver as full cDNA expression constructs. This novel engineering methodology allowed for high level *COL7A1* expression in allogeneic umbilical cord blood hematopoietic stem cells and peripheral blood T-cells. This robust and user-friendly platform is readily deployable for targeted gene overexpression in multiple applications.

Gene therapy using exogenous gene encoding regulatory elements can result in supraphysiological gene expression in autologous or allogeneic cells. This approach can be highly effective particularly in disease phenotypes where therapeutic peptide(s) produced from a circulating depot population of cells can facilitate systemic therapeutic benefit. A crucial determinant for efficient gene therapy-based cellular engineering is the choice of delivery vehicle which can be broadly categorized into viral and non-viral systems that either integrate into the genome or are maintained as extrachromosomal species. The size of the gene is a key consideration for the choice of vector as many systems are restricted by the limits of cargo they can effectively accommodate and deliver. Larger cargo (5+ kb open reading frames) are generally more difficult to deliver and can be associated with higher rates of toxicity.

Non-integrating expression vectors can be maintained as a non-integrating episome that in cells and tissues with a low proliferative capacity can be maintained long term. However, ex vivo modification of cells with limited growth potential is challenging due to scalability and therapeutic dose of cells able to be delivered. Integrating vectors result in permanent insertion into the genome and long-term expression in parental and progeny cells making them well suited for sustained supranormal gene expression. There is an offsetting risk; however, of genomic sequence disruption and potential for insertional mutagenesis. A further important consideration for gene therapy applications is the potential for adverse immunological side effects. The use of autologous cells that are educated in the host environment represents a desirable approach for minimizing the occurrence of transplant associated complications. A major hurdle to the realization of autologous therapies is efficient and uniform ex vivo engineering of patient cells. The numbers of cells and frequency of gene transfer can be highly variable such that complete therapeutic benefit may not be achieved. Certain disorders such as RDEB that have large open reading frames make delivery of the therapeutic cargo a compounding challenge. Thus, there is a gap in the field of gene and cellular therapy for generating a standardized population of cells with a low propensity for immune-based side effects that express supraphysiological levels of a candidate gene in a manner that maintains the integrity of the genome. To address this deficiency, we instituted a line of study to determine the optimal conditions for candidate gene expression using the native gene with targeted introduction of powerful gene activation components upstream of the start codon. We hypothesized that lymphohematopoietic cells, due to their broad circulatory potential and relative ease of obtainment, would be an ideal population for engineering. We tested our hypothesis in umbilical cord blood hematopoietic stem cells and peripheral blood T-cells. These cells are of high translational impact and have a favorable innate or acquired immunological profile for broad transplant application. UCB hematopoietic progenitors have a lowered risk of causing graft versus host disease allowing for greater donor:recipient mismatches in the allogeneic transplant setting. T-cells, through a secondary engineering step to ablate the T-cell receptor complex, can be rendered inert in regard to antigen recognition capability. In these cells we optimized the engineering parameters for gene expression upregulation for the generation of a uniform pool of cells as an off the shelf product with the potential to be given to multiple recipients with a low risk of allogeneic transplant associated side effects.

The emergence of easily produced programmable nucleases such as the CRISPR/Cas9 system have allowed for precision gene targeting with a specificity that greatly mitigates collateral “off target” genomic damage. CRISPR/Cas9 is comprised of two components: the Cas9 nuclease and a small guide RNA transcript. The functional complex of Cas9 and the gRNA bind the target DNA sequence and generates a single or double stranded DNA break that can be repaired from an exogenous repair template by the error free homology directed repair DNA pathway. While gene editing represents a transformative strategy for cellular engineering, it has been limited to date in its ability to uniformly produce therapeutic levels of gene expression. Editing-based gene correction has centered on two strategies: site specific repair or incorporation of a full open reading frame coding sequence and regulatory elements at safe harbor loci. The correction of a disease-causing base in situ results in maintenance of gene expression from the endogenous regulatory elements that may not drive gene expression levels to the therapeutic threshold. Alternatively, safe harbor locus incorporation of an exogenous promoter, candidate cDNA, and polyadenylation signal can drive high level gene expression. The most common safe harbor is the *AAVS1* locus on chromosome 19 that has served as what could be termed a landing pad for user defined open reading frame/regulatory element sequence incorporation. However, the ability to incorporate genes in excess of five kilobases into this locus in therapeutically relevant cells can be challenging. Therefore, we hypothesized that targeted knock in of transcriptional elements and a reporter gene upstream and in frame with an endogenous gene would facilitate gene upregulation and selection of modified cells.

To determine the optimal position to insert the UMET element we screened three guide RNA candidates in a ~200 bp window immediately upstream of the native *COL7A1* transcriptional start site (Figure 1A). One candidate showed demonstrable activity as assessed by Surveyor assay and sequencing (Figure 1C,D). To confirm that this candidate would be suitable for promoting gene activity, we introduced a nuclease inactive Cas9 that retains DNA binding ability and is fused to a VP64 transcriptional activator [25]. Using this system, we observed a ~6-fold increase in gene expression in HEK 293T cells, demonstrating that this portion of the *COL7A1* locus was favorable for promoting transcriptional upregulation (Figure 1E). Importantly, this version of Cas9 is >5 kb making it difficult to deliver. Our design strategy with the UMET targeting construct represents a novel and streamlined approach for candidate gene upregulation.

Because few reports show UCB HSC modification with CRISPR/Cas9, we rigorously optimized the conditions for HDR in this cell population that possesses a greater ability to be delivered to disparate patients in allogeneic transplant. The donor units of HSCs routinely showed a high degree of CD34^+^CD133^+^ cells that are characteristic of HSCs (Figure 2A). The Cas9 delivery format was then assessed for HDR ability, efficiency, and kinetics. Comparisons between Cas9 mRNA and Cas9 recombinant peptide were performed using a homologous recombination reporter system that is well characterized for the *AAVS1* safe harbor locus (Figure 2B) [26]. It is designed to express GFP only upon HDR-based insertion into the first intron of the *PPP1R12C* locus. Delivery of the donor was accomplished by AAV-6 transduction, a serotype shown to be highly effective at transducing CD34^+^ HSCs [32]. We observed that a Cas9:gRNA ribonucleoprotein complex was more efficient at facilitating HDR when the AAV-6 particles were added in a narrow time window after electroporation (Figure 2C). The analyses in Figure 2C were performed three days after gene transfer and to rigorously assess the timing of HDR post-electroporation and immediate AAV-6 donor addition, we screened cells at defined time points. Based on the data in Figure 2D we show that the cells undergo HDR modification in a ~24-h time period. This narrow window in which editing occurs is of tremendous importance as it allows for the ex vivo/in vitro culture time to be streamlined in order to maintain the phenotype of the stem cell population. The optimal dose of donor was next assessed and we observed that a dose of 5 × 10^5^ was capable of mediating robust HDR (Figure 2E). The CRISPR/Cas9 modified cells were then placed into semi-solid methycellulose for colony forming unit potential. There were no differences between control, unmodified cells and those that underwent Cas9 RNP electroporation, AAV-6 transduction, and *AAVS1* locus HDR (Figure 2F). These data demonstrated that the optimized gene editing conditions did not perturb HSC function and lineage commitment in vitro.

Using the parameters defined in Figure 2 we then designed, built, and tested a novel donor (UMET) for targeted *COL7A1* gene upregulation. It consisted of a UCOE sequence that has been shown to maintain an open chromatin profile [28], the MND promoter that is well defined for strong activity in T-cells and HSCs [29], and the tEGFR that serves for both selection and downstream considerations for safety by allowing for targeted depletion as a suicide gene. The tEGFR was placed in frame with the endogenous *COL7A1* gene by virtue of a T2A sequence (Figure 3A). The entire targeting cassette was ~4 kb to allow for efficient AAV packaging. Cas9 RNP electroporation followed by donor addition resulted in ~10% HSC gene modification rates that allowed for recovery of a pure population of tEGFR^+^ CD34^+^ cells for molecular and phenotypic characterization (Figure 3B). Inside out PCR showed *COL7A1* locus HDR (Figure 3C) and qRT-PCR showed that donor incorporation drove a 15-fold increase in *COL7A1* expression in HSCs (Figure 3D). The modified cells showed both a normal distribution and morphology of hematopoietic lineage derivatives in vitro demonstrating that supraphysiological *COL7A1* expression did not impact CFU potential (Figure 3E,F). These data show that *COL7A1* locus targeting and gene expression did not appreciably affect the phenotype and properties of the HSCs. While a diminished rate of *COL7A1* gene targeting compared to *AAVS1* was observed, we propose that this is related to the differential locus accessibility to the CRISPR/Cas9 reagents. The *COL7A1* locus is not predicted to be highly active in HSCs and thus may be condensed with chromatin making it less amenable to targeting than *AAVS1*. It is for these reasons we included a UCOE element in our design in order to promote greater gene expression in areas of the genome with diminished transcriptional levels. Importantly, our targeting efficiencies are in line with previous HSC gene editing experiments and the unique inclusion of the tEGFR allows for cell isolation and, paired with scaling, the engineering process is viable for downstream (e.g., clinical) application(s). Collectively, our defined engineering conditions observations are crucial for clinical scale up and application and our optimized protocol promotes a rapid turnaround time for HSC engineering and delivery.

Toward further defining the optimal donor platform we generated a truncated UCOE sequence and a donor that only had a promoter (Figure 4A). These donors were tested in primary T-cells and gene targeting frequency was measured by tEGFR expression. The lowest rates of HDR were seen in the donor with the minimal UCOE element while the MND only and UCOE MND-based donors were nearly equivalent (Figure 4B). The levels of tEGFR expression remained stable over two weeks with significant cell expansion over that period (Figure 4C). To assess the properties of these modified cells we purified them by magnetic bead isolation for tEGFR expression and performed molecular and protein analysis. In highly purified cells (Figure 5A) a fusion transcript using inside/outside PCR primers was observed showing co-expression of tEGFR and *COL7A1* (Figure A3). Quantification of *COL7A1* mRNA expression showed that in the purified cell populations the *COL7A1* expression levels were equivalent across the donor candidates (Figure 5B). Comparison to wild type keratinocytes showed that the engineered T-cells produce more *COL7A1* mRNA than this major *COL7A1* producing cell population. Western blot analysis demonstrated that the T-cells were capable of producing higher levels of *COL7A1* protein than keratinocytes and that the peptide adopted the proper architecture as evidenced by collagenase digestion (Figure 5C,D). Next, in order to make the T-cells broadly applicable we ablated the T-cell receptor such that they lose their ability to cause GVHD (Figure 5E). Importantly, this strategy has been used clinically for immunotherapy [33].

The cell choices for our study are carefully considered: UCB HSCs represent a potential lifelong source of *COL7A1* and represent a putative advance over current bone marrow transplant that infuses and engrafts cells with physiological levels of *COL7A1*. T-cells could be considered for augmentative or shorter-term treatment options given that they may circulate for a short time, deliver the payload, and then slowly decline due to effector cell exhaustion. To realize the potential for this approach our T-cell engineering design included TCR disruption such that they cannot initiate GVHD. As such, both of the cell populations have a favorable immunological profile with a potential as an off the shelf product for delivery to multiple patients.

Our strategy is reliant on AAV-based delivery of the donor and this size restricted vector is ideal for facilitating HDR. A recent human genome level screen showed that Cas9 mediated upregulation of target genes occurs within a ~200 bp window proximal to the transcriptional start site [25]. This is highly significant given that our UMET (UCOE.MND.tEGFR.t2A) construct allows for 500 bp of homology arm sequence making our operational capacity within range of every gene in the human genome. We also observed that the MND promoter only version mediated similar expression levels to UMET over a 14-day time course (Figure 4). This adds to the amount of donor sequence that can be included in the targeting arms and provides expanded flexibility under circumstances/in models where the targeting window may exceed 500 bp.

Our studies provide proof of principle in cells with high translational impact. Importantly, the design is intended to be flexible and expansive in its application with potential for cellular engineering in support of translation, production, and manufacturing. In conclusion, our robust platform represents a broadly applicable tool for gene upregulation with direct application in diverse models, organisms, and gene and cellular expression systems.

## 4. Materials and Methods

### 4.1. Human Cell Sample Purification

Primary T-cells and umbilical cord blood derived CD34 cells were obtained in accordance with the Declaration of Helsinki requirements for research on human subjects with approval of the University of Minnesota Institutional Review Board IRB# 0305M47681. T-cells were isolated using the RosetteSep Human T Cell Enrichment Cocktail (STEMCELL Technologies, Cambridge, MA, USA) and the CD34 MicroBead kit (Miltenyi Biotec, Auburn, CA, USA) was used to enrich for CD34^+^ cells from cord blood. EGFR positive selection was accomplished by adding 3.0 µg/mL of phycoerythrin (PE) labeled anti-human EGFR antibody (Biolegend, San Diego, CA, USA; Clone AY13) and the EasySep PE positive selection kit (STEMCELL Technologies, Cambridge, MA, USA) Human embryonic kidney 293T cell line were purchased from ThermoFisher (Waltham, MA, USA).

### 4.2. Culture Conditions

293T cells were maintained in Dulbecco’s Modification of Eagle’s Medium supplemented with glutamax, non-essential amino acids, penicillin/streptomycin and 10% fetal bovine serum ThermoFisher (Waltham, MA, USA). Cells were maintained at 37 °C and 5% CO_2_.

T-cells were grown in X-VIVO-20 (Lonza, Allendale, NJ, USA) with 10% AB serum (Valley Biomedical, Winchester, VA, USA), 300 IU of IL-2 and 5 ng/mL each of IL-7 and IL-15 (PeproTech, Rocky Hill, NJ, USA), *N*-Acetyl-l-Cysteine, penicillin/streptomycin, and Gluta-MAX-I each from ThermoFisher (Waltham, MA, USA).

CD34^+^ hematopoietic stem cells were cultured in StemSpan SFMII media containing 1 µM SR1 each from STEMCELL Technologies, Cambridge, MA, USA and human cytokines Flt-3 ligand (100 ng/mL), SCF (100 ng/mL), TPO (100 ng/mL), IL-6 (100 ng/mL) all from Biolegend, San Diego, CA, USA.

### 4.3. CRISPR/Cas9

Guide RNAs were obtained from Synthego (Redwood City, CA, USA) and were used at a concentration of 1 μg with 10 μg of Cas9 protein (Aldevron, Fargo, ND, USA) or 1 μg of mRNA (TriLink, San Diego, CA, USA). Guide RNA target sequences (5′-3′) were:

*COL7A1-1:* GGCAGUAAAAGCCGUCAGCU
*COL7A1-2:* GCGGACGCGCAGGCAAGACC
*COL7A1-3:* AGAAAAGUCCCUGAUCUCGG
*TRAC:* GAGAAUCAAAAUCGGUGAAU
*AAVS1:* GUCACCAAUCCUGUCCCUAG

### 4.4. Gene Transfer

Plasmids for guide RNA candidate testing were delivered via Lipofectamine 2000 (ThermoFisher, Waltham, MA, USA) to 293 cells in cis using the Guide-IT CRISPR/Cas9 system (Clontech, Mountain View, CA, USA) T-cells were activated with CD3/CD28 Dynabeads (ThermoFisher, Waltham, MA, USA) at a 3:1 bead to cell ratio. Six hours prior to gene transfer the beads were removed. Electroporation was performed using the Neon Electroporation System (ThermoFisher, Waltham, MA, USA) with 10 μL tips using buffer T. T cell Neon settings: 1400 volts, 10 ms, 3 pulses. HSC Neon settings: 1450 volts, 10 ms, 3 pulses. Cells were plated in anti-biotic free media for 12–24 h.

GFP mRNA was obtained from TriLink BioTechnologies (San Diego, CA, USA). AAV-6 particles were produced by Vigene (Rockville, MD, USA) and were added at the indicated MOI and AAV particle units are genome copies (GC)/mL. *COL7A1* upregulation testing was performed using gRNA 3 and in 293T cells using dCAS9-VP64-GFP that was a gift from Feng Zhang (Addgene plasmid # 61422).

### 4.5. Molecular Analysis

293T cells treated for 72 h with the candidate nuclease and the *COL7A1* target locus sequence was amplified with F: 5′-TGGTCACTGTGATTGACCTAAA-3′ and R: 5′-GGAGTTGGCTGGGTTGT-3′ at 94C 2 min and 40 rounds of 94C 40 s, 58C 40 s, 68C 1 min, and a final extension of 68C for 10 min. Surveyor assay was performed using the Surveyor Mutation Detection Kit (Integrated DNA Technologies, Coralville, IA, USA) with resolution on a 10% polyacrylamide gel.

Inside/out HDR PCR. Genomic DNA was amplified using AAVS1 F: 5′-GGACGAGCTGTACAAGTAACG-3′ and R: 5′-GAGACAGTGACCAACCATCC-3′ or tEGFR F: 5′-CAGTGTGCCCACTACATTGA-3′ and *COL7A1* R: 5′-TGAGGAGCCATCCAGTAAGA-3′ using Phusion High-Fidelity DNA Polymerase (New England BioLabs, MA, USA) with the following conditions: 98C × 30 s and 35 cycles of 98C × 10 s, 63C × 15 s, and 72C × 30 s.

Amplicons were either directly sequenced (Sequetech, Mountain View, CA, USA) or TA cloned (ThermoFisher, Waltham, MA, USA) and then sequenced.

Inference of CRISPR Edits (ICE). Sanger files were analyzed for insertions and deletions using the ICE algorithim (https://ice.synthego.com/#/; Synthego, Redwood City, CA, USA).

Quantitative reverse transcription PCR. Total RNA was reverse transcribed with SuperScript VILO (ThermoFisher) and analyzed by TaqMan gene expression assay using the 2^−∆∆*C*T^ method. The *COL7A1* probe was Hs00164310_m1 and the normalization control was *GAPDH* Hs99999905_m1 (ThermoFisher).

### 4.6. Western Blot

Cell lysates were resuspended in RIPA buffer (MilliporeSigma, Burlington, MA, USA). Undigested or collagen digested (via 37C incubation for 2 h at 37 °C with collagenase from Worthington Biochemical Co., Freehold, NJ, USA) were resolved on a Tris-Acetate gel under reducing conditions. GAPDH was the loading control (MilliporeSigma, Burlington, MA, USA) and collagenase samples were probed with an anti-C7 antibody that was a kind gift from Dr. David Woodley and Dr. Mei Chen. Full length C7 protein was analyzed with an anti-human antibody (Abnova, Walnut, CA, USA). Image densitometry was performed with ImageJ, (National Institutes of Health, Bethesda, MD, USA).

### 4.7. Colony Forming Assay

Ten thousand tEGFR purified HSCs were placed in MethoCult semi-solid media (STEMCELL Technologies, Cambridge, MA, USA). At day 14 the colonies were enumerated and scored for morphology by an experienced, blinded reviewer.

### 4.8. Flow Cytometry

FACS data was acquired on a BD LSRII Cytometer (Becton Dickinson, Franklin Lakes, NJ, USA) and data was analyzed with FlowJo 10.4.2 (FlowJo, LLC, Ashland, OR, USA). The following antibodies were used: CD34: Alexa Fluor 488 clone 581; CD133 PE-Dazzle Clone clone 7; CD3 APC/Cy7 clone: OKT3; Zombie Violet^TM^ Fixable Viability Kit all from BioLegend (San Diego, CA, USA).

### 4.9. Images

Design images are power point templates from Motiflio (Ellicott City, MD, USA)

## 5. Conclusions

We show here the development and characterization of a powerful and versatile platform for targeted gene activation that will be a valuable tool for the genome and cellular engineering communities.

## Abbreviations

CRISPR	clustered regularly interspaced short palindromic repeats
C7	type VII collagen
TALEN	transcription activator like effector nucleases
HDR	homology directed repair
UCB	umbilical cord blood
HSC	hematopoietic stem cells
GVHD	graft versus host disease
gRNA	Guide RNA
indel	insertions/deletions
ICE	Inference of CRISPR Edits
RNP	ribonucleoprotein
MOI	multiplicity of infection
CFU	colony forming unit
AAV	Adeno-associated virus
SA	splice acceptor
UCOE	ubiquitous chromatin opening element
tEGFR	truncated epidermal growth factor receptor gene
UMET	UCOE.MND.tEGFR.T2A
NC	non-collagenase domain
KC	keratinocytes

## Appendix E. DNA Sequences

*COL7A1* left homology arm:

ATGACATTCTCTCCTCCTTCCGGCTTATCCCCTACAAAGAGGGGGTGAGTTACTTGGATCCAGGCCAAGGGGACCTTGGTTTCCCTAAGACCGGCCCAGAGTCACTCATTTGCCAGGGCTTCTTGCCTGTCAAGGAGATCCGGGTGGGGCCCAGGAGGCCCACCAGACAGATGGCTGAATCACAGGAGTGGCCGGCGGGACCCATGGCCTGAGGGCTTGTCTGGGCACCCCCACTGGATTGGGGGTGAGTCATCCCCAACTGCAGCCCCACCCCCCACGGCGCTGCTGCCTTGTGGCTCTGCAGGAACCTGTCCACTCCTCAGCCTGGTCACTGTGATTGACCTAAAGCAGCCAAGACCTGTGACCTTAGATGGAGTTAGGGGTACTCCCTCAGCATCTGCCCATGCAGAACCTTCTGGGAAATTCCCAGAAGCCACGGGGGGTCGGGGGGTTTATAGTTAAGTGCGTCATATCGTTTGTCTGGGGGAGGGGTGGGGGGGGCGGCGACCTCTCAGGGATATGGGTGAGGGCGGGTGCCTGGGTTCCCGCCTGCCGCTCCGCCC

*COL7A1* Right homology arm:

ACGCTGCGGCTTCTGGTGGCCGCGCTCTGCGCCGGGATCCTGGCAGAGGCGCCCCGAGTGCGAGCCCAGCACAGGGAGAGAGGTGGGCACCGCAAGGGAGGACCCGGCCCGGGGCACTGCACCGTGCCACCCTCCGCTCCACTCGGCCTTCATCCCCAACACCCCCCGCCCACAACCCAGCCAACTCCACGACGCCCCGCGGATTCCTCCTAATTCTGGGACTCCCCGAACCCTCCGCGATCGGTGCTGGGTTCCCTAAACCGCCTCCTATCCTGTTCCTACCCAAATTCTGGCTCCCTGGATGCGTGCGGGGTCCCCTGCCTTATGCCAATCCACGCCGGCCCCTAGAGGCTGACCTCAGTCCCAAGTCCACACCCGTGCTGGCGACTCTCCCTCCCCCGCACACCCCCAACCCTCCGCGGGAGTCTGCTCTGGGACCCACCGAGCTCAACTCCCTCCAAGGCCCTCAGGCTCAGGCCGATTCGGCCCTGGTTTGCATAAGATGAGGCCTACTCTCCCCACCATCCCAAGTCCCAGTGAGGCCACCGTGCGG

T2A sequence:

GAGGGCAGAGGAAGTCTTCTAACATGCGGTGACGTGGAGGAGAATCCCGGCCCT

tEGFR:

ATGCTTCTCCTGGTGACAAGCCTTCTGCTCTGTGAGTTACCACACCCAGCATTCCTCCTGATCCCACGCAAAGTGTGTAACGGAATAGGTATTGGTGAATTTAAAGACTCACTCTCCATAAATGCTACGAATATTAAACACTTCAAAAACTGCACCTCCATCAGTGGCGATCTCCACATCCTGCCGGTGGCATTTAGGGGTGACTCCTTCACACATACTCCTCCTCTGGATCCACAGGAACTGGATATTCTGAAAACCGTAAAGGAAATCACAGGGTTTTTGCTGATTCAGGCTTGGCCTGAAAACAGGACGGACCTCCATGCCTTTGAGAACCTAGAAATCATACGCGGCAGGACCAAGCAACATGGTCAGTTTTCTCTTGCAGTCGTCAGCCTGAACATAACATCCTTGGGATTACGCTCCCTCAAGGAGATAAGTGATGGAGATGTGATAATTTCAGGAAACAAAAATTTGTGCTATGCAAATACAATAAACTGGAAAAAACTGTTTGGGACCTCCGGTCAGAAAACCAAAATTATAAGCAACAGAGGTGAAAACAGCTGCAAGGCCACAGGCCAGGTCTGCCATGCCTTGTGCTCCCCCGAGGGCTGCTGGGGCCCGGAGCCCAGGGACTGCGTCTCTTGCCGGAATGTCAGCCGAGGCAGGGAATGCGTGGACAAGTGCAACCTTCTGGAGGGTGAGCCAAGGGAGTTTGTGGAGAACTCTGAGTGCATACAGTGCCACCCAGAGTGCCTGCCTCAGGCCATGAACATCACCTGCACAGGACGGGGACCAGACAACTGTATCCAGTGTGCCCACTACATTGACGGCCCCCACTGCGTCAAGACCTGCCCGGCAGGAGTCATGGGAGAAAACAACACCCTGGTCTGGAAGTACGCAGACGCCGGCCATGTGTGCCACCTGTGCCATCCAAACTGCACCTACGGATGCACTGGGCCAGGTCTTGAAGGCTGTCCAACGAATGGGCCTAAGATCCCGTCCATCGCCACTGGGATGGTGGGGGCCCTCCTCTTGCTGCTGGTGGTGGCCCTGGGGATCGGCCTCTTCATG

UCOE:

ATAACTGAGAGAAAAAAATCCAGAAAGCTGGATTTAATACAAAGTAAAAAAGGTAAGCTTCGCACAGTCATTAGCTGTTATTATAAACACCTTCATAGTCAGCTGCAGAGTGGAGACTGAGTATTTACAGTATATTATTAAAAGCAAAGCAGGAAATGTTGTACTCAGAAATATTACTAACAACTTTTTTTCAGCCAGGTGCAGTGGCTGTTGCCTGTAATCCCAGCGCTTTGGGAGGCTGAGGTCAGAGAATCACTTGAGCCAAGGAGTTCAAGACCAGCCTGACCAACATAGGGAGACCCTGTCTCTACAAACAATTTAAAAATTAGCCAAGTGTAGTGGCATATACCTTCTACTCTAGAGGCAGAGGTAGGAGAGTTGCTTGAGCTCAGGAGGTCAAGGCTGCAGTGAGCTGAGATCACGCCACTGCATTCTAGCCTTGGTGACAGAGCAAGACCCTGTCTCACACATAAAAAAAGAAAAAGTATTTTTTATGGCCGGGCATGGTTGCTCATGCCTATAATCCCAGCACTTTGGGAGGCTGAGAGGGGAGGATCACTTGAGGTCAGGAATTCAAGACCAGCCTGGCCAACATGGCAAAGCCCTGCTCTTGGCCGGGCACAGTGGCTTATGCCTATAATCCTAGCACTTTGGGAGGCAAAGGTGGGTGGATTGCTTAAGCCCAGTAATTTGAGACCAGCTTGGGCAGTGTGGCGAAACCTCATCCCTACAAAAAATACAAAAACTTAGCTGGGTATGGTGGTGTGTGCCTGTAATCCCAGCTACTTGGGAGGCTGAGGTGGGAGGATCACATGAACCTGGGAGGCAGAGGCTACAGTGAGCTGATTGGGCCACTGCACTCCAGCCTGGACAACAGAGTGAGACCATGCCTCAAAAACAAAACAAAACCCTTCTATTAGGCAGCTTTTGTGTGTGTGTGTCTCGCTGTGTTGCCCAAGCTGGGGTGCAACTGTGCAACTTTGGCCCACTGCAGCCTACATCTCCTGGTTCAAGCAATTCTCGTGCCTCAGCGTCCTGAGTAGCTGGGATTACAGGCGTGAGCCACCATGCTTGGACTTTTTTTTTTTTGAAACAGGATCTCACTTTTTTGCTCAGGCTGGAGTGCAATGGCATGATCAAGGCTCACTGCACCCTCAACCTCCCTGGGCTCAATCAGTCCTTCTACCTCAGCCTCCCTAGTAGCTGAGACTAC

Minimal UCOE:

GGGAGGTGGTCCCTGCAGTTACGCCAATGATAACCCCCGCCAGAAAAATCTTAGTAGCCTTCCCTTTTTGTTTTCCGTGCCCCAACTCGGCGGATTGACTCGGCCCCTTCCGGAAACACCCGAATCAACTTCTAGTCAAATTATTGTTCACGCCGCAATGACCCACCCCTGGCCCGCGTCTGTGGAACTGACCCCTGGTGTACAGGAGAGTTCGCTGCTGAAAGTGGTCCCAAAGGGGTACTAGTTTTTAAGCTCCCAACTCCCCCTCCCCCAGCGTCTGGAGGATTCCACACCCTCGCACCGCAGGGGCGAGGAAGTGGGCGGAGTCCGGTTTTGGCGCCAGCCGCTGAGGCTGCCAAGCAGAAAAGCCACCGCTGAGGAGACTCCGGTCACTGTCCTCGCCCCGCCTCCCCCTTCCCTCCCCTTGGGGACCACCGGGCGCCACGCCGCGAACGGTAAGTGCCGCGGTCGTCGGCGCCTCCGCCCTCCCCCTAGGGCCCCAATTCCCAGCGGGCGCGGCGCGCGGCCCCTCCCCCCGCCGGGCGCGCGCCCGCTGCCCCGCCCTTCGTGGCCGCCCGGCGTGGGCGGTGCCACCCCTCCCCCCGGCGGCCCCGCGCGCAGCTCCCGGCTCCCTCCCCCTTCGGATGTGGCTTGAGCTGTAGGCGCGGAGGGCC

MND Promoter:

ATCGATCACGAGACTAGCCTCGAGAAGCTTGATATCGAATTCCACGGGGTTGGACGCGTCTTAATTAAGGATCCAAGGTCAGGAACAGAGAAACAGGAGAATATGGGCCAAACAGGATATCTGTGGTAAGCAGTTCCTGCCCCGGCTCAGGGCCAAGAACAGTTGGAACAGCAGAATATGGGCCAAACAGGATATCTGTGGTAAGCAGTTCCTGCCCCGGCTCAGGGCCAAGAACAGATGGTCCCCAGATGCGGTCCCGCCCTCAGCAGTTTCTAGAGAACCATCAGATGTTTCCAGGGTGCCCCAAGGACCTGAAATGACCCTGTGCCTTATTTGAACTAACCAATCAGTTCGCTTCTCGCTTCTGTTCGCGCGCTTCTGCTCCCCGAGCTCTATATAAGCAGAGCTCGTTTAGTGAACCGTCAGATCGCCTGGAGACGCCATCCACGCTGTTTTGACCTCCATAGAAGACACCGACTCTAGAGGATCGATCCCCCGGGCTGCAGGAATTCAAGCGAGAAGACAAGGGCAGAAAGCACC
